# Study protocol for testing pharmacological conditioning as a drug dose reduction strategy in patients with psoriasis in a randomised controlled trial

**DOI:** 10.1136/bmjopen-2025-114026

**Published:** 2026-04-15

**Authors:** Paraskevi Savvidou, Stefanie Hölsken, Frederik Krefting, Sven Benson, Wiebke Sondermann

**Affiliations:** 1 University of Duisburg-Essen, Institute for Medical Education, Center for Translational and Behavioral Neurosciences, University Hospital Essen, Essen, Germany; 2 University of Duisburg-Essen, Department of Dermatology, Venereology and Allergology, University Hospital Essen, Essen, Germany; 3 University of Duisburg-Essen, Institute of Medical Psychology and Behavioral Immunobiology, Center for Translational and Behavioral Neurosciences, University Hospital Essen, Essen, Germany; 4 Department of Dermatology, Uniklinikum Erlangen, Friedrich-Alexander University Erlangen-Nürnberg, Erlangen, Germany; 5 Uniklinikum Erlangen, Deutsches Zentrum Immuntherapie (DZI), Erlangen, Germany

**Keywords:** Psoriasis, Quality of Life, Randomized Controlled Trial

## Abstract

**Introduction:**

Psoriasis is a chronic inflammatory skin disease that significantly impacts patients’ quality of life. Although biological therapies are effective, they are associated with high costs and potential side effects, necessitating strategies for dose reduction. Pharmacological conditioning, using placebo mechanisms through associative learning, presents a promising approach to maintain therapeutic efficacy with lower doses of medication.

**Methods and analysis:**

The single-centre, randomised controlled trial aims to investigate pharmacological conditioning with secukinumab in patients with moderate-to-severe psoriasis (N=168). Participants will be randomly allocated to a treatment-as-usual group or one of two experimental groups receiving partial or continuous reinforcement schedules with reduced secukinumab doses combined with a distinctive gustatory stimulus. Primary outcomes include changes in itch intensity, skin-related quality of life and objective disease severity. Secondary outcomes encompass psychological variables, side effects and biological markers. Results may contribute to optimised long-term psoriasis management, reducing medication burden while maintaining treatment efficacy.

**Ethics and dissemination:**

The study protocol was approved by the ethics committee of the University Hospital Essen (19–8636 BO) on 20 November 2023. Written informed consent will be obtained from all participants. Participant confidentiality will be ensured through pseudonymised data handling and secure storage. The results will be disseminated through peer-reviewed publications.

**Trial registration number:**

DRKS00034977.

**STRENGTHS AND LIMITATIONS OF THIS STUDY**

- The longitudinal design (10 assessments) enables modelling of dynamic symptom trajectories in addition to pre–post change.
- The comparison of two dose-reduction regimens against each other and against standard dosing informs individualised treatment strategies.
- The merging of the two conditioning groups allows equalising sample sizes for the non-inferiority test, whereas statistical power may be limited for arm-specific comparisons with treatment-as-usual.
- With no low-dose, non-conditioned controls, pharmacological low-dose effects cannot be disentangled from conditioning effects.

## Introduction

 Psoriasis is a chronic inflammatory skin disease affecting approximately 2–3% of the global population and is associated with burdening psychosocial comorbidities including depression, anxiety disorders and suicidal ideation.^1^,^3^ Psoriasis is a lifelong relapsing and remitting condition that significantly impairs patients’ quality of life, as it is often accompanied by physical discomfort, impaired emotional functioning, a negative body- and self-image and limitations in daily activities.^4^

Over the last decade, treatment options for psoriasis have massively expanded. Advanced systemic therapies now include the latest generation of biologics, specifically targeting pro-inflammatory cytokines identified as pivotal in pathophysiology of psoriasis such as interleukin (IL)-17 and IL-23. These modern cytokine blockers have demonstrated high efficacy in alleviating symptoms in patients with moderate-to-severe psoriasis.^5^ However, biologics impose a significant economic burden and carry potential side effects, which can negatively affect patients’ adherence to treatments.^5^,^10^ As a result, dosage reduction strategies were proposed as a possibility to optimise treatment efficacy while minimising risks and healthcare costs.^11^

Although reviews have summarised existing clinical studies on dose tapering of biologics in psoriasis,^11 12^ most studies investigated stepwise dose reduction based on clinical stability criteria, without reference to psychological mechanisms or behavioural frameworks. However, recent research suggests that such mechanisms may offer a promising avenue for enhancing symptom relief beyond the pharmacological action of the drug itself.^13^ Placebo effects, traditionally considered a confounding factor in clinical trials, may in fact be intentionally used for therapeutic purposes.

A large body of evidence indicates that treatment expectations, as a core psychological mediator of placebo effects, significantly influence both the efficacy and tolerability of pharmacological treatments across a wide range of clinical conditions.^14^,^20^ This is particularly relevant in dermatology, where clinical studies have demonstrated that skin diseases and allergic reactions can be modulated through various methods of inducing placebo effects, such as enhancing positive treatment expectations or using implicit learning mechanisms like pharmacological conditioning.^16 20 21^ Given that placebo effects can induce measurable symptom relief independent of active pharmacological agents, their systematic integration into psoriasis treatment could serve as a complementary strategy to help sustain symptom control despite lower biological dosages. This would optimise treatment outcomes while reducing costs and potential side effects.

One particularly promising target for such an approach in psoriasis patients is itch, which affects approximately 85% of patients and represents a core symptom of psoriasis.^22^ Beyond its physical manifestation, itch significantly impacts patients’ quality of life, contributing to emotional distress, impaired daily functioning and reduced overall well-being.^23^ Notably, itch is highly susceptible to psychological influences,^16 20^ as cognitive and emotional factors can modulate its perception and intensity. A key mechanism underlying this susceptibility is the skin-brain axis, a bidirectional communication system linking psychological states to immune and inflammatory processes in the skin.^24 25^ This makes itch a particularly promising target for placebo-based interventions and further supports the rationale for harnessing placebo effects in psoriasis treatment. Accordingly, placebo effects on itch are routinely observed in the treatment of various dermatological conditions, including psoriasis.^2021 26^,^28^ Clinical and experimental studies have demonstrated that these placebo effects are mediated by treatment expectations and implicit learning processes.^16 20^ While these findings highlight the general susceptibility of itch to placebo mechanisms, most work has focused on acute placebo effects induced by verbal suggestions, which were shown to induce conscious positive treatment expectations,^29^,^31^ but show limited efficacy in sustaining long-term symptom control, which is particularly relevant for chronic conditions such as psoriasis.^32^ Preliminary evidence from a recent randomised controlled trial conducted by our research group supports the notion that verbal suggestions alone may not be sufficient to generate sustained placebo effects in chronic inflammatory conditions such as psoriasis.^33^ In this trial (N=120), patients with moderate-to-severe psoriasis received the IL-17A antagonist secukinumab and were assigned to one of three groups: standard treatment (300 mg), a dose-reduction group (75 mg) and an experimental group receiving the same reduced dose along with verbal suggestions aimed at enhancing positive treatment expectations. While all groups showed symptom improvement over time, verbal suggestions did not significantly reduce subjective itch, quality of life or objective symptom scores compared with dose reduction alone, suggesting limited efficacy of conscious expectation modulation.

Given these limitations, approaches that use implicit learning processes—such as pharmacological conditioning—may offer a more effective means of sustaining symptom relief in chronic diseases like psoriasis. Unlike verbal suggestions, which primarily induce conscious expectations, pharmacological conditioning engages implicit learning processes, in which repeated pairing of an active drug with a neutral stimulus (eg, the appearance or intake of a medication) leads to the conditioned stimulus alone eliciting a physiological response similar to that of the drug.^13 34^ This approach has shown potential in dermatology, as demonstrated by evidence from experimental itch research suggesting that conditioning-based placebo effects are more robust and longer-lasting than those induced by verbal suggestions alone, especially when both mechanisms are combined.^35^,^37^

A proof-of-concept study by Ader and colleagues^38^ performed in patients with mild-to-moderate psoriasis provides preliminary clinical evidence for the efficacy of pharmacological conditioning in psoriasis treatment. In a partial reinforcement schedule, active corticosteroids were intermittently replaced with a placebo after an acquisition phase with full dose medication administration. Results indicated a greater amelioration of symptoms and less relapse for partial reinforcement when compared with a continuous reinforcement schedule with the same cumulative amount of the drug.^38^ To date, this study remains the only one which investigated pharmacological conditioning as a dose-reduction strategy in the treatment of psoriasis. Nevertheless, the study highlights the potential of personalised reinforcement schedules as an adjunct to standard psoriasis treatment.

Building on the promising findings of Ader and colleagues, our present study aims to expand on this preliminary evidence by investigating the effects of pharmacological conditioning as a dose-reduction strategy in systemic psoriasis treatment with the IL-17 inhibitor secukinumab. To further optimise associative learning and enhance response durability, the study intentionally selects the conditioned stimulus based on general principles derived from experimental evidence. Specifically, data suggest that the success of pharmacological conditioning depends critically on the characteristics of the conditioned stimulus. To maximise learning efficiency, the conditioned stimulus should be perceptually distinct and highly associable with the pharmacological effect.^13^ Prior research on preparedness has shown that certain sensory stimuli—particularly gustatory cues—are more readily linked to immune-related responses, making them particularly well-suited for conditioning paradigms involving psoriasis.^39^ Additionally, the salience and uniqueness of a drug, including its appearance, taste and mode of administration, have been found to influence the strength and persistence of conditioned responses.^40 41^ To systematically address these factors, our study employs a well-established green-coloured, novel-tasting drink (strawberry milk aromatised with lavender oil and dyed with green food dye) as described previously as a conditioned stimulus, designed to optimise associative learning processes and enhance response durability.^42^

By investigating pharmacological conditioning as a dose-reduction strategy in the systemic treatment of patients with moderate-to-severe psoriasis, this approach may offer a novel strategy to improve long-term disease management while minimising medication burden.

## Methods

### Trial design

To evaluate the efficacy of pharmacological conditioning as a dose-reduction strategy for patients with moderate-to-severe psoriasis, a single-centre, non-inferiority study with a randomised factorial design will be conducted at the Department of Dermatology, University Hospital Essen, Germany.

**Figure 1:**
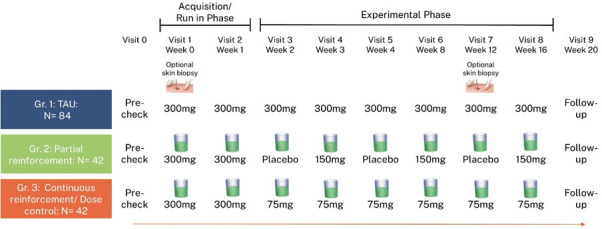
Dosing schedule at a glance. TAU, treatment-as-usual.

In order to assess non-inferiority of pharmacological conditioning compared with standard treatment, eligible patients will be randomly assigned in a 2:1:1 allocation ratio to one of three study arms: treatment-as-usual (TAU), partial reinforcement and continuous reinforcement, respectively. Patients in the TAU group will receive standard pharmacological treatment with full doses of the monoclonal antibody secukinumab, serving as the active control group. Patients in the partial reinforcement group will receive a reduced dose of secukinumab or a placebo at alternating visits, while those in the continuous reinforcement group will receive reduced doses of secukinumab throughout all visits. To disentangle potential dose effects from conditioning effects, the continuous reinforcement group serves as a dose-control group with identical cumulative dosage to the partial reinforcement group. This enables the evaluation of whether partial reinforcement schedules elicit stronger clinical effects than continuous reinforcement, despite equal drug exposure. In both reinforcement groups, a novel-tasting beverage will be administered in combination with the medication or placebo to elicit pharmacological conditioning. For an overview of the dosing schedule, see figure 1. The study was preregistered on 18 October 2024 at the German Clinical Trials Register (DRKS00034977) prior to the first recruitment on 13 December 2024 and is conducted as part of the Collaborative Research Center 289, which investigates the impact of patient expectations on the efficacy of medical treatments (https://treatment-expectation.de/en/). Recruitment is planned to continue until 30 June 2027. This study protocol adheres to the SPIRIT 2025 statement (Standard Protocol Items: Recommendations for Interventional Trials). The completed SPIRIT checklist is provided as online supplementary table 2.^43^

### Funding information

This work is funded by the Deutsche Forschungsgemeinschaft (DFG, German Research Foundation), https://www.dfg.de/; Project-ID 422744262 - TRR 289, to WS and SB). The funders have no role in study design; collection, management, analysis and interpretation of the data; writing of the report; or the decision to submit the report for publication.

### Inclusion criteria

Patients eligible for the trial must meet the following criteria: a minimum age of 18 years, an indication for the initiation of a systemic biological therapy according to the German S3-guideline on psoriasis,^44^ a Psoriasis Area and Severity Index (PASI) score >10. All sexes are eligible for trial participation.

### Exclusion criteria

Patients are not eligible if they meet any of the following criteria: pregnancy, lactation, forms of psoriasis other than chronic plaque (eg, pustular, erythrodermic, guttate or drug-induced) psoriasis, insufficient comprehension and communication skills in the German language, inability to provide informed consent, contraindications to biological therapy with secukinumab, medical or psychiatric conditions that contraindicate therapy with secukinumab or participation in the study, ongoing use of systemic antipsoriatic treatments, as well as prior treatment with secukinumab.

### Study schedule

Patients will attend a total of 10 visits over 20 weeks, beginning with an eligibility screening prior to a baseline assessment (visit 0), followed by a 16-week treatment phase (visits 1–8) and a follow-up assessment at week 20 (visit 9). All study visits will take place in the clinical study centre of the Department of Dermatology, University Hospital Essen, Germany, and will be conducted by the study physician, research assistants and medical student assistants.

#### Recruitment and enrolment of patients

Patients will be recruited from the outpatient department and from the inpatient wards of the Department of Dermatology, Venereology and Allergology at the University Hospital Essen, Germany. Recruitment will be conducted directly by the treating physician, who will inform patients with moderate-to-severe psoriasis about the option to participate in a study evaluating the influence of psychological factors on the effectiveness of systemic therapy with the IL-17 inhibitor secukinumab on subjective and objective outcomes. Additionally, informational flyers about the study for referring dermatologists will be included in the patients’ discharge letters.

Interested patients will be invited to a clinical visit (visit 0) at the study outpatient clinic, where they will be asked to provide written informed consent for participation by the treating study physician. Patients will be informed that they will be randomly assigned to one of three groups testing different dosing protocols for secukinumab, with some groups receiving lower doses than the standard regimen.

Additionally, patients will be informed that, depending on group assignment, they will receive a novel-tasting beverage alongside their injections at each visit. The patient information further includes a timeline indicating when psychological variables and biological samples will be collected. It will be emphasised that group allocation will be disclosed at the end of the study and that all patients will have access to standardised treatment with secukinumab or another therapeutic option after the study.

Patients will be fully informed about potential side effects, their right to withdraw their consent for the study at any time and the confidentiality of their data. On enrolment, each participant will be assigned a unique pseudonym through the ALIIAS software.^45^ The pseudonym will be linked to the web-based survey platform LimeSurvey,^46^ which will be used to collect the survey data for the study.

#### Dosing schedule and administration

Patients in the TAU group (n=84) will receive the standard dosing regimen of 300 mg of secukinumab as weekly injections for 5 weeks (weeks 0–4), followed by 3 monthly injections in weeks 8, 12 and 16. The injections will be administered by the research assistant to ensure that the study physician remains blinded to group allocation. Patients in both reinforcement groups will initially receive weekly injections of 300 mg of secukinumab during an acquisition phase (weeks 0–1). After the acquisition phase, patients in the partial reinforcement group will receive alternating weekly injections of 150 mg of secukinumab or a placebo (ie, sodium chloride) for 3 weeks (weeks 2–4) followed by monthly injections in weeks 8, 12 and 16. Injections for the patients in the continuous reinforcement group will be administered in the same weekly and monthly schedule as in the other experimental group but will consist of a continuous dose of 75 mg of secukinumab. Both reinforcement groups will receive the same total amount of medication to ensure that any observed effects will be attributable to the pharmacological conditioning, rather than differences in the total doses of the medication in the TAU and partial reinforcement group.

Additionally, at each visit, patients in both reinforcement groups will be given a novel-tasting gustatory stimulus (ie, 100 mL of fruit milk dyed with green food colouring and infused with lavender oil) right before medication or placebo administration. The research assistant will conduct the administration of the beverage, accompanied by standardised information about its potential benefits via a medical educational video, which was developed in collaboration with the Collaborative Research Center’s (CRC) patient advisory board.

#### Baseline assessment (visit 0)

Prior to the start of the treatment phase, patients will complete a baseline assessment during visit 0. After providing written informed consent, they will first complete a battery of psychological self-report questionnaires. This will be followed by a clinical consultation with the study physician, during which objective symptom severity will be assessed using the PASI. In addition, blood samples will be collected to monitor physiological parameters and for subsequent cytokine analysis. Saliva samples will also be collected for cortisol level analysis during this visit. Optionally, a skin biopsy will be obtained prior to the administration of the first dose of medication (ie, before visit 1).

#### Treatment phase and follow-up assessment

##### Visit 1

Similar to the procedure at visit 0, patients will start by completing psychological self-report questionnaires, followed by the clinical assessment conducted by the study physician. In contrast to visit 0, no biological samples will be collected. For patients assigned to one of the reinforcement groups, a 2-minute-35-second long medical educational video will be shown. The video will refer to a novel tasting green beverage, which, when taken in conjunction with the medication, can increase its effect via conditioning processes. Patients will be informed that they will receive the beverage immediately prior to their medication at every visit throughout the treatment phase. A literal transcription of the original German script as well as an English translation is provided as supplementary material (online supplemental text 1 and 2). The administration of the green beverage prior to the medication administration will be performed as the final step of each visit during the treatment phase.

##### Visit 2–visit 8

Procedures during visits 2 and 8 will be identical to those at visit 1, with the exception that additional blood samples will be collected during visits 5 and 8. An optional second skin biopsy will be collected prior to visit 7 to explore longitudinal changes in response to treatment.

##### Visit 9 (follow-up)

The procedure during the follow-up visit will be similar to that of the treatment phase, but will not include medication administration or biological sample collection.

### Patient adherence and study discontinuation

To improve adherence to the intervention protocol, all medication or placebo injections will be administered by the research assistant at the clinical study centre, which requires the attendance of patients. Similarly, all questionnaire data will be assessed prior to the medication administration on site.

Given the relatively low dosages of secukinumab in the reinforcement groups, topical treatments will be permitted as concomitant care in all three groups to enhance adherence and reflect real-life clinical care, as recommended by the S3 guideline for the treatment of psoriasis vulgaris. The study participation will be discontinued at any time at the patient’s request, if side effects are deemed intolerable by the patient and/or the treating physician or if the patient misses more than one visit. In such cases, the patient will be unblinded to the treatment allocation to allow appropriate subsequent medical care.

### Outcomes

#### Primary outcomes

##### Itch

The change in itch severity will be assessed as primary outcome throughout the intervention and follow-up phase. Participants will be asked to rate the most severe itch as well as the average itch experienced over the last 24 hours on a Visual Analogue Scale (VAS). The scale ranges from 0 (‘no itch’) to 100 (‘most severe itch imaginable’). Specifically, participants will be asked: ‘How severe was the most intense itch you experienced in the last 24 hours?’ (German: ‘Wie stark war der schlimmste Juckreiz, den Sie in den letzten 24h gespürt haben?’) and ‘How severe was your average itch in the last 24 hours?’ (German: ‘Wie stark war Ihr durchschnittlicher Juckreiz in den letzten 24h?’). This measurement will be carried out during visits 1–9.

##### Skin-related quality of life

Skin-related quality of life will be measured by the Dermatology Life Quality Index (DLQI),^47^ a validated self-report measure that quantifies subjective quality of life in adults with skin conditions such as psoriasis vulgaris. Patients will be asked to rate symptom severity and impairment across various areas of life over the past 7 days on a scale from 0 (‘not at all’) to 3 (‘very much’). This measurement will be conducted at all visits. As with itch, quality of life was selected as a primary outcome to serve as a meaningful subjective indicator for symptom alleviation.

### Secondary outcomes

#### Objective symptom severity

The PASI^48^ will be used as a clinical scoring tool to assess the extent of affected body surface area and the severity of key symptoms in patients with psoriasis. As the most commonly used clinical tool for objectively evaluating psoriasis severity,^48^ the PASI provides a composite score ranging from 0 to 72, with scores of at least 10 representing a moderate-to-severe degree of psoriasis severity. The PASI will be conducted at all visits.

#### Psychological secondary outcomes

Online supplemental file 1 provides an overview of the time points and the respective questionnaires used. These outcomes are also collected as part of the CRC’s central projects, which coordinate and administrate standardised collection of additional measures across all study sites and subprojects involving human participants for merged analyses. According to the CRC’s approach, these measures will serve as additional analyses aiming to identify predictors of expectation effects.

##### Average Stress (VAS)

Patients will rate their average stress using the item ‘How stressed have you felt on average in the last 24 hours?’ (German: ‘Wie gestresst haben Sie sich in den letzten 24h im Durchschnitt gefühlt?’) on a VAS from 0 (‘no stress at all’) to 100 (‘greatest imaginable stress’).

##### Expected skin-related quality of life after the study (DLQI-Expectations)

To assess the expected skin-related quality of life after the study, patients will be asked to rate modified items from the DLQI^47^ on a 5-point-Likert scale from 0 (‘not at all’) to 4 (‘very much’). The content of the items mirrors the items of the original questionnaire, with the exception of asking for subjective anticipations of the skin-related quality of life after study participation (‘How much do you think your skin disease will affect your life at the end of our study?’; German: ‘Was denken Sie, wie sehr Ihre Hauterkrankung Ihr Leben am Ende unserer Studie beeinflussen wird?’). An overall score of expected skin-related quality of life will be calculated as the sum of all individual values across all items. Expected skin-related quality of life will be assessed at four time points (visits 1, 5, 7 and 9).

##### Itch (ecological momentary assessment)

To complement visit-based assessments of itch, real-time itch intensity will be measured using ecological momentary assessment (EMA). Current itch will be assessed by asking patients: ‘How severe is your itch at the moment?’ (German: ‘Wie stark ist Ihr Juckreiz in diesem Moment?’). Patients will respond on a VAS ranging from 0 (‘no itch’) to 100 (‘most severe itch imaginable’). Assessments will be delivered via the smartphone application m-Path,^49^ which will be preprogrammed with fixed-time prompts. Participants will receive notifications at 08:00, 14:00 and 20:00 on the respective days before and after each study visit, as well as on the day of the visit itself. A non-randomised time sampling approach was selected to reduce participant burden and enhance compliance across the extended study period. Given the high prevalence of itch in psoriasis patients and its significant impact on quality of life, itch was chosen to reflect a meaningful subjective indicator of symptom alleviation. Its susceptibility to psychological influences also characterises changes in itch as a useful marker for assessing the impact of psychological expectation effects on symptom severity in the context of pharmacological conditioning.

##### Patient Benefit Index

Subjective treatment needs and their achievement will be assessed using the 25-item Patient Benefit Index (PBI).^50^ Participants will rate the subjective value of need prior to treatment at baseline measurement point and the extent to which each treatment need was met during the treatment on a 6-point Likert scale from 0 (‘not at all’) to 5 (‘very much’). Needs prior to treatment and needs achieved during treatment will be converted to a weighted index score. The PBI will be assessed at five time points (visits 0, 3, 5, 7 and 9).

##### Patient global assessment

The patient global assessment (PGA)^51^ will be used to assess the current perceived severity of psoriasis symptoms on a 5-point Likert scale from 0 (‘no psoriasis/normal skin’) to 4 (‘severe psoriasis’). The PGA will be measured at nine time points (visits 1–9). Additionally, a modified version of the PGA will be used. As with the modified DLQI, the item mirrors the item of the original questionnaire with the exception of assessing subjective expectations of symptom severity after study participation (‘What severity of psoriasis do you expect at the end of our study?’; German: ‘Welche Schwere Ihrer Psoriasis (Schuppenflechte) erwarten Sie am Ende unserer Studie?’). The modified PGA will be assessed at four time points (visits 1, 5, 7 and 9).

##### Patient’s perception of the conditioned stimulus

To assess how feasible, effective and valuable the patients perceived the novel beverage in enhancing the drug’s effect, they will rate three items on a VAS ranging from 0 (‘not at all’) to 100 (‘completely’). Specifically, patients will be asked ‘How pleasant is it for you to drink the beverage?’ (German: ‘Wie angenehm ist es für Sie, das Getränk zu trinken?’), ‘How effective do you find the drink in increasing the effectiveness of your medication?’ (German: ‘Wie effektiv finden Sie das Getränk, um die Wirksamkeit Ihres Medikaments zu steigern?’) and ‘How useful do you find the drink to help your body fight psoriasis?’ (German: ‘Wie nützlich finden Sie das Getränk, um Ihren Körper dabei zu unterstützen, gegen die Schuppenflechte zu kämpfen’). The patient’s perception of the conditioned stimulus will be assessed at three time points (visits 5, 7 and 9).

##### State-Trait Anxiety Inventory

The short version of the State-Trait Anxiety Inventory (STAI)^52^ will be used to measure state anxiety. Patients will rate their current anxiety on a scale from 0 (‘not at all’) to 4 (‘very much’). A total score for state anxiety will be calculated as the sum of individual values across all items. The STAI will be assessed at nine time points (visits 1–9).

##### Attribute Influences on Perceived Treatment Effects (VAS)

To assess the degree to which patients attribute perceived treatment effects to potential influencing factors, they will be asked to respond to the item: ‘To what extent do you think the following factors will have an influence on your treatment?’; German: ‘Was denken Sie, in welchem Maße die folgenden Faktoren einen Einfluss auf Ihre Behandlung haben werden?’). Patients will rate this item for four potential treatment influences: the physician, themselves, the medication and the conditioned stimulus (with the conditioned stimulus only included for patients assigned to a reinforcement group), using a VAS ranging from 0 (‘no influence’) to 100 (‘greatest possible influence’). Treatment influences will be assessed at two time points (visits 2 and 9).

##### Treatment Satisfaction (Ecological Momentary Assessment, EMA)

To capture real-time data on treatment satisfaction, patients will answer an EMA item at 8:00 PM on the day of each visit (‘How satisfied are you with your therapy at the moment?‘; German: ‘Wie zufrieden sind Sie im Moment mit Ihrer Therapie?‘). Patients will rate the item on a VAS ranging from 0 (‘no satisfaction’) to 100 (‘total satisfaction’).

### Biological secondary outcomes

#### Blood samples

During visits 0, 5 and 8, blood samples will be collected to monitor physiological responses to the medication, enable cytokine analysis and ensure the safety of systemic therapy.

#### Saliva samples

During visit 0, saliva samples will be collected using the Oragene-DNA OG-600 kit (DNA Genotek, Ottawa, Canada). Additionally, patients will be instructed to collect saliva samples for analysis of awakening cortisol and alpha-amylase on two consecutive days after visit 0. The samples will be collected right after waking up, as well as 30 and 45 min after awakening. Patients will be informed that saliva collection should take place prior to food or coffee consumption. To facilitate the saliva collection, the research assistant will set up an app on the patients’ smartphones. The app will provide acoustic reminders to ensure timely collection of the saliva samples. Additionally, the app will guide patients to track the sample collection by scanning barcodes on the salivettes. Patients will be instructed to return the salivettes at their next study visit. Samples will be used to assess the cortisol awakening response as part of the CRC’s central projects.

#### Skin biopsies

As an optional measure, patients can provide consent for the collection of skin biopsies. The collected skin samples will be used to assess changes in biomarkers of psoriasis before and after 12 weeks of therapy. At visit 1 and visit 7, two 4 mm punch biopsies will be taken from the consenting patients: one from psoriasis-affected skin and one from unaffected skin, resulting in a total of four biopsies per participant. A total of 10 patients per group are planned to provide skin biopsies.

### Moderators

As part of the CRC’s comprehensive, standardised questionnaire battery, the following moderator variables will be assessed:

#### Beck Depression Inventory

Patients will rate their severity of depression using the Beck Depression Inventory (BDI-II)^53^ on a 4-point-Likert scale from 0 (‘no symptoms’) to 3 (‘severe symptoms’). An overall score for depression will be computed as the sum of individual values across all items. The BDI-II will be measured at three time points (visits 1, 5 and 9).

#### Big-Five Inventory

To assess the Big-5 personality traits, patients will rate the items of the Big-Five Inventory (BFI-10)^54^ on a 5-point Likert scale from 1 (‘does not apply at all’) to 5 (‘fully applicable’). Mean scores for each personality subscale will be calculated. The BFI-10 will be measured once at visit 0.

#### Desire for Itch Relief (VAS)

Patients will rate their desire for itch relief using the item ‘How much would you like your itching to improve as a result of the treatment?’ (German: ‘Wie sehr wünschen Sie sich, dass sich Ihr Juckreiz durch die Behandlung bessert?’) on a VAS from 0 (‘not at all’) to 100 (‘greatest possible desire’). Desire for itch relief will be assessed once at visit 1.

#### Generic Assessment of Side Effects

The Generic Assessment of Side Effects (GASE)^55^ will be used to assess the frequency and severity of various side effects and their attribution to the study medication. Participants will rate the severity of side effects on a scale from 0 (‘not present’) to 3 (‘severe’) and will indicate whether they attributed the side effects to the study medication using a dichotomous item (‘yes’/‘no’). General symptom load will be calculated as the sum of all symptom ratings, while a medication-attributed total score will be calculated as the product of the individual item answers and the binary attribution rating (1 = ‘yes’; 0 = ‘no’). The GASE will be measured at nine time points (visits 1–9).

#### Generic Rating Scale for Previous Treatment Experiences, Treatment Expectations and Treatment Effects

Patients will rate their previous treatment experiences at baseline as well as treatment expectations and acute treatment effects during the treatment phase using the GEEE (Generic Rating Scale for Previous Treatment Experiences, Treatment Expectations and Treatment Effects)^56^ on a 10-point-Likert scale from 0 (‘none’) to 10 (‘greatest imaginable’). Previous treatment experiences and treatment expectations will each be measured once, at visit 0 and visit 1, respectively. Treatment effects will be assessed at eight time points (visits 2–9).

#### Interoceptive Awareness and Sensitivity Questionnaire

Patients will rate items from the Interoceptive Awareness and Sensitivity Questionnaire (ISAQ)^57^ on a 5-point Likert scale from 1 (‘strongly disagree’) to 5 (‘strongly agree’). An overall score for all three subscales (ie, sensitivity to neutral bodily sensations, attention to unpleasant bodily sensations, difficulty disengaging from unpleasant bodily sensations) will be computed as sum of all individual values across the items of each subscale. The ISAQ will be assessed once at visit 0.

#### Menstrual cycle assessment

A short questionnaire will be used to assess menstrual cycle characteristics. All patients will be asked whether they currently menstruate and whether they consider themselves premenopausal, perimenopausal or postmenopausal. If participants report currently menstruating and being premenopausal, they will be additionally asked to provide information on the first day of their most recent menstrual period, the average length of their menstrual period (in days) and whether they are currently using any hormonal contraceptive, with a prompt to specify the type used. Menstrual cycle characteristics will be assessed once at visit 1.

#### Pain Disability Index

The Pain Disability Index (PDI)^58^ measures the extent to which pain affected the participants’ ability to function in various areas of life. Participants will rate their level of pain disability on a 10-point Likert scale from 0 (‘no disability’) to 10 (‘total disability’) across seven life domains. A total score for pain disability will be calculated as the sum of all item ratings. The PDI will be assessed at two time points (visits 0 and 9).

#### Perceived Stress Scale

As part of the Perceived Stress Scale (PSS),^59^ patients will be asked to rate the frequency of stress-related thoughts and feelings over the past month on a 6-point Likert scale from 0 (‘never’) to 5 (‘very often’). A total score for perceived stress will be calculated as the sum of the individual values across all items. The PSS will be assessed once at visit 0.

#### Somatosensory Amplification Scale

Patients will rate their increased sensitivity to bodily sensations using the Somatosensory Amplification Scale (SSAS)^60^ on a 5-point-Likert scale from 1 (‘completely’) to 5 (‘not at all’). An overall score will be calculated by summing the individual values across all items. The SSAS will be assessed once at visit 0.

#### State-Trait Anxiety Depression Inventory-state

State anxiety and state depression will be assessed using the State-Trait Anxiety Depression Inventory (STADI-state).^61^ Patients will be instructed to rate the intensity of their current anxiety and depression symptoms on a 4-point Likert scale from 1 (‘not at all’) to 4 (‘very much’). An overall score will be calculated as the sum of the individual values across all items. The STADI-state will be measured at nine time points (visits 1–9).

#### STADI-trait

Trait anxiety and trait depression will be assessed using the STADI-trait.^61^ Patients will be instructed to rate the frequency of anxiety and depression-related symptoms on a 4-point Likert scale from 1 (‘rarely’) to 4 (‘very often’). An overall score will be calculated as the sum of the individual values across all items. The STADI-trait will be measured once at visit 0.

#### Substance use assessment

Substance use will be assessed using a questionnaire that captures the frequency and quantity of alcohol, nicotine and caffeine consumption over the past 7 days. Specifically, patients will be asked: ‘On how many days and in what quantity have you consumed one of the following substances in the past week?’ (German: ‘An wie vielen Tagen und in welcher Menge haben Sie in der vergangenen Woche eine der folgenden Substanzen konsumiert?’). For each substance, patients will be asked to indicate the number of days on which they had consumed the substance during the past week and the total number of units consumed during that time frame. A standardised unit definition will be provided: one unit of alcohol will be defined as 0.33 L of beer, 0.15 L of wine or 0.04 L of spirits. One unit of cigarettes will be defined as one cigarette, and one unit of caffeine will be defined as one cup of coffee, black or green tea or 0.5 L of coca cola.

In addition, patients will have the option to report any further psychoactive substances they had used within the time frame. Substance use will be assessed once at visit 1.

#### Treatment Expectation Questionnaire

To assess various dimensions of treatment expectations, patients will rate items from the Treatment Expectation Questionnaire (TEX-Q)^62^ using a VAS ranging from 0 (‘none’) to 10 (‘greatest imaginable’). An overall treatment expectation score will be calculated by averaging the ratings across all items. To assess specific dimensions of treatment expectation (eg, probability, valence and outcome), a sum score will be computed for each subscale. The TEX-Q will be assessed once at visit 1.

#### Warmth and competence

To assess the perceived warmth and competence of the treating physician, patients will rate items of the Warmth and Competence Scales of the Stereotype Content Model^63^ on a 5-point-Likert scale from 1 (‘not at all’) to 5 (‘extremely’). Mean scores for subscales will be calculated. Warmth and competence will be assessed once at visit 9.

### Sample size, group allocation and blinding

#### Sample size

To test the non-inferiority of the drug-dose reduction strategy to the standard care at an exploratory level, the sample size calculation was conducted in R using the epiR package (function: epi.ssninfb). This calculation is based on a non-inferiority framework using the absolute risk difference scale. For the purpose of calculation, a favourable outcome was assumed for 70% of participants in the reinforcement group and 75% in the TAU group. The non-inferiority margin was set to an absolute risk difference of 0.25, corresponding to a minimal clinically important difference (MCID) of >25%. Assuming a one-sided α=0.05 and 80% power, a total sample size of N=152 was required for a 1:1 comparison between TAU and the pooled reinforcement groups. As the primary non-inferiority hypothesis compares TAU with the pooled reinforcement groups, the sample size calculation was based on a 1:1 allocation ratio.

To account for the dropout rate of 10% during a previous study, which investigated the impact of verbal inductions of positive treatment-related expectations as a dose-reduction strategy in patients with psoriasis,^33^ a sample size of N=168 is set.

#### Group allocation

The allocation of patients to the study groups will take place after enrolment and receipt of written informed consent. To balance disease severity between groups, the randomisation of patients will follow a stratified randomisation schedule based on PASI scores and will be carried out by the research assistant. The stratified randomisation schedule will be generated prior to study initiation by a postdoctoral researcher who is not involved in patient care, recruitment or data collection. The study physician will have no access to the randomisation list. Prior to recruitment, two strata were established based on PASI scores: 60% of patients were expected to have a PASI ≤15 and 40% a PASI of >15. Within each stratum, randomisation to one of the three intervention groups will be performed using computer-generated random numbers. These numbers will be generated using an Excel spreadsheet and will determine the respective group assignments. To ensure blinding, the group assignment will be initially blackened out, with the group only revealed after the assignment has been completed.

#### Blinding

The study uses a double-blind design, where both the patients and the study physicians will be blinded with regard to the group allocation of patients. The blinding process will be managed by the research assistant, who will administer medication/placebo and the novel tasting beverage. During the administration of medication and placebo, respectively, patients will be instructed to wear a sleep mask to prevent them from seeing the medication. Additionally, the width of the syringes will be standardised to 27-gauge for both the secukinumab and placebo injections, ensuring that patients will not be able to distinguish between the injected substances. Blinding will be implemented to prevent biased interpretations of objective symptom severity by the study physician and to avoid any negative treatment expectations caused by knowledge of the reduced medication dosage or placebo administration.

### Patient and public involvement statement

Patients and the public were not involved in the development of the research questions, outcome measures, study design, recruitment or conduct of the study. Further, patients were not involved in assessing the burden of the intervention or the time required to participate in the study. However, a patient advisory board was consulted prior to the start of the study to evaluate the medical educational video used within the intervention. Dissemination of the study results to healthcare professionals and the general public will be conducted as part of the overarching communication strategy of the CRC 289 ‘Treatment Expectation’.

### Ethics and dissemination statement

The study protocol was approved by the ethics committee of the University Hospital Essen (19–8636 BO) on 20 November 2023. Written informed consent will be obtained from all participants. Participant confidentiality will be ensured through pseudonymised data handling and secure storage. The results will be disseminated through peer-reviewed publications.

### Data management

All study data will be collected electronically during on-site visits using tablet devices. Standardised psychometric questionnaires were programmed and will be implemented via the platform LimeSurvey.^46^ Data collected via LimeSurvey will be automatically pseudonymised using the ALIIAS software.^45^ These pseudonymised data will be transferred to the AutoCRF infrastructure, which performs automated checks for completeness and plausibility. Raw and processed data will be securely stored in the project-specific directories within the centralised infrastructure of the collaborative research centre. Only authorised study personnel will have access to these data.

Printed and electronic versions of patient consent forms and contact information will be stored separately at the outpatient clinic, with access restricted to authorised personnel only.

### Analysis plan

All statistical analyses will be conducted using R^64^ (V.4.1.1.; R Core Team, Vienna, Austria) within RStudio^65^ (RStudio Team, Boston, MA).

Confirmatory hypotheses will be tested using linear mixed models, assuming approximate normality of random effects and residuals. If model diagnostics indicate substantial violations of distributional assumptions, generalised linear mixed models with appropriate link functions and distribution will be applied based on the observed characteristics of the data.

Each model will include Group as a between-subject factor and Time (10 levels, one per visit) as a within-subject factor. The Group factor represents the treatment conditions (TAU, partial reinforcement and continuous reinforcement). The number of Group levels varies by hypothesis and will be specified within each hypothesis section. Random intercepts for participants will be included to account for individual differences. Random slopes will be added if they significantly improve model fit, as evaluated by likelihood ratio tests and information criteria (ie, Akaike Information Criterion, Bayesian Information Criterion).

Main effects and Group × Time interactions will be examined depending on the respective hypotheses. Pairwise comparisons of estimated marginal means will be conducted where appropriate, with Holm-adjusted p values used to control for multiple comparisons, particularly given the unbalanced group sizes. A significance level of α=0.05 will be applied for all confirmatory tests.

#### Hypothesis 1

We hypothesise that both experimental treatments (pooled across partial and continuous reinforcement) will be non-inferior to the TAU group regarding average reductions in itch, PASI and DLQI scores from pre-measurement to post-measurement. Non-inferiority testing will be embedded within the linear mixed model framework. One-sided 95% CIs will be computed based on model estimates. Non-inferiority will be concluded if the upper bound of the CI lies below the predefined MCID of 25%.^66^

#### Hypothesis 2

We hypothesise that symptom trajectories differ between groups. Specifically, both experimental groups are expected to show slower improvements in objective disease severity (ie, PASI scores), while improvements in subjective symptoms (ie, itch and DLQI scores) are expected to be comparable to those in the TAU group. Group × Time interactions will be modelled to identify differential symptom trajectories. Given the absence of prior data on the exact shape of these trajectories, no assumptions about their functional form are made beyond the directionality stated above. Post hoc contrasts will be conducted to explore group differences at specific time points or in change patterns across the study period.

#### Hypothesis 3

As part of hypothesis 3, we examined potential effects on secondary outcomes, namely side effects. Specifically, we hypothesise that participants in the experimental groups (pooled across partial and continuous reinforcement) will report fewer side effects from pre to post compared with the TAU group. A linear mixed model with a Group × Time interaction will be fitted to side effect scores derived from the GASE questionnaire.

#### Hypothesis 4

Supplementary to the other hypotheses, a subsequent analysis focusing on the two reinforcement groups will examine potential differences in both overall and longitudinal effects of the various dosing regimens. We hypothesise that the partial reinforcement group will show stronger treatment effects on outcomes compared with the continuous reinforcement group. Specifically, we expect significantly greater average reductions in itch, PASI and DLQI scores over time. This hypothesis will be tested via the Group × Time interaction across all 10 measurement points. If the interaction is significant, follow-up contrasts will compare the estimated change trajectories between the two experimental groups, with a focus on overall pre–post improvement and the rate of change over time.

### Exploratory analyses

In addition to the confirmatory analyses, exploratory analyses will be conducted to examine potential associations between the primary outcomes and various secondary and moderating variables, including demographic characteristics (eg, age, gender), psychometric measures (eg, depression, anxiety), biological markers (ie, blood, saliva) and ecological momentary assessments (ie, itch ratings, treatment satisfaction). These analyses are exploratory in nature and are intended to generate hypotheses for future research.

### Missing data handling

All primary analyses will follow the intention-to-treat (ITT) principle, including all patients who received at least one active dose of the study medication and completed the acquisition phase (ie, attended visits 0–2). Patients meeting this criterion will be included in the analysis with all available data up to the point of dropout. By contrast, individuals who only attended the baseline assessment (visit 0) will be excluded from longitudinal modelling due to the absence of any post-baseline intervention exposure (ie, no medication or conditioned stimulus was administered). Missing data will be addressed using Full Information Maximum Likelihood (FIML) as the primary estimation approach. FIML leverages all available data points, including partially observed cases, by estimating model parameters based on the observed-data likelihood, without imputing missing values. This method is particularly suitable for complex mixed-effects models and preserves statistical power while minimising bias under the assumption of data missing at random (MAR).

Given the structured nature of data collection, where all assessments are completed on-site prior to medication administration, only a low proportion of missing data is expected. Reasons for missed visits (eg, illness, psychological burden) will be documented systematically through physician–patient contact and follow-up communication. In addition, a wide range of psychometric, demographic and clinical variables (eg, depression, anxiety, acute stress, treatment satisfaction) is collected, which increases the possibility that missingness is conditionally random (ie, MAR) and can be accounted for by observed data.

To empirically evaluate the MAR assumption, we will conduct logistic regression analyses to identify variables that significantly predict the occurrence of missing data. If no such variables are found, the originally specified analysis model will be estimated using FIML, without the inclusion of auxiliary covariates, thereby ensuring consistency with the pre-specified hypothesis structure.

However, if significant predictors of missingness are identified, multiple imputation (MI) will be employed as a complementary strategy. In this case, auxiliary variables will be included in the imputation model only, while the analysis model will remain unchanged. This allows for the modelling of potential MAR mechanisms without introducing auxiliary covariates into the hypothesis-driven analysis, thereby preserving the interpretability of the predefined main and interaction effects.

To ensure sufficient information for reliable imputation, only participants with at least 7 out of 10 observed time points (ie, ≤30% missingness) will be included in the MI procedure. Participants with fewer than 7 observed time points will not be imputed but will remain in the FIML-based analyses in line with the ITT principle. In these cases, parameters will be estimated solely based on the likelihood of the available observations, without extrapolation.

A comparison between estimates obtained via FIML and MI will serve as a sensitivity analysis, evaluating the robustness of results under different plausible missing data assumptions, while maintaining a clear separation between hypothesis testing and missing data handling.

## Supplementary material

10.1136/bmjopen-2025-114026 online supplemental file 1

10.1136/bmjopen-2025-114026 online supplemental file 2

10.1136/bmjopen-2025-114026 online supplemental file 3
