# Supplementary material for: Study protocol for testing pharmacological conditioning as a drug dose reduction strategy in patients with psoriasis in a randomised controlled trial
Source: BMJ Open. 2026 Apr 15;16(4):e114026. doi: 10.1136/bmjopen-2025-114026 (PMC13084905; doi:10.1136/bmjopen-2025-114026)
Supplement: online supplemental file 1 [file bmjopen-16-4-s001.docx]

**Supplementary Table 1.** Overview of time points and collected self-report data.
At all survey time points, an unblinded research assistant collected patients’ data on the electronic study tablet.

| **Time Point (Visit)** | **Assessed self-report questionnaires** |
| --- | --- |
| t1 (Visit 0) | - Demographic characteristics - Prior treatment experiences (GEEE) - Trait anxiety and depression (STADI-Trait) - Stress (PSS) - Sensitivity and awareness of bodily sensations (SSAS) - Personality traits (BFI-10) - Skin-related quality of life (DLQI) - Pain disability (PDI) - Interoceptive awareness and sensitivity (ISAQ) - Treatment goals (PBI) - Prior treatment experiences for psoriasis |
| t2 (Visit 1) | - Menstrual cycle characteristics - Nicotine and alcohol consumption - Treatment expectations (GEEE; TEX-Q) - State anxiety and depression (STADI-state; BDI-II; STAI short) - Skin-related quality of life (DLQI) - Itch (visual analogue scale) - Physical ailments at baseline (GASE) - Acute and expected skin-related quality of life after study (DLQI) - Acute and expected severity of psoriasis after study (PGA) - Itch (visual analogue scale; ecological momentary assessment) - Stress (visual analogue scale) - Treatment satisfaction (ecological momentary assessment) - Qualitative Interviews & Single Category Implicit Association Test |
| t3 (Visit 2) | - State anxiety and depression (STADI-state) - Skin-related quality of life (DLQI) - Itch (visual analogue scale) - Side effects (GASE) - Acute treatment effects (GEEE, PGA) - Subjective predictors for acute treatment effects - Stress (visual analogue scale) - State anxiety (STAI short) - Itch and treatment satisfaction (ecological momentary assessment) |
| t4 (Visit 3) | - State anxiety and depression (STADI-state) - Skin-related quality of life (DLQI) - Itch (visual analogue scale) - Side effects (GASE) - Acute treatment effects (GEEE, PGA) - Stress (visual analogue scale) - State anxiety (STAI short) - PBI - Itch and treatment satisfaction (ecological momentary assessment) |
| t5 (Visit 4) | - State anxiety and depression (STADI-state) - Skin-related quality of life (DLQI) - Itch (visual analogue scale) - Side effects (GASE) - Acute treatment effects (GEEE, PGA) - Stress (visual analogue scale) - State anxiety (STAI short) - Itch and treatment satisfaction (ecological momentary assessment) |
| t6 (Visit 5) | - State anxiety and depression (STADI-state) - Depression (BDI-II) - Skin-related quality of life (DLQI) - Itch (visual analogue scale) - Side effects (GASE) - Acute treatment effects (GEEE, PGA) - Expected skin-related quality of life after treatment (DLQI) - Expected symptom severity after treatment (PGA) - Stress (visual analogue scale) - State anxiety (STAI short) - PBI - Patient´s Perception on the conditioned stimulus - Itch and treatment satisfaction (ecological momentary assessment) |
| t7 (Visit 6) | - State anxiety and depression (STADI-state) - Skin-related quality of life (DLQI) - Itch (visual analogue scale) - Side effects (GASE) - Acute treatment effects (GEEE, PGA) - Stress (visual analogue scale) - State anxiety (STAI short) - Itch and treatment satisfaction (ecological momentary assessment) |
| t8 (Visit 7) | - State anxiety and depression (STADI-state) - Skin-related quality of life (DLQI) - Itch (visual analogue scale) - Side effects (GASE) - Acute treatment effects (GEEE, PGA) - Expected skin-related quality of life after treatment (DLQI) - Expected symptom severity after treatment (PGA) - Stress (visual analogue scale) - State anxiety (STAI short) - PBI - Patient´s Perception on the conditioned stimulus - Itch and treatment satisfaction (ecological momentary assessment) |
| t9 (Visit 8) | - State anxiety and depression (STADI-state) - Skin-related quality of life (DLQI) - Itch (visual analogue scale) - Side effects (GASE) - Acute treatment effects (GEEE, PGA) - Stress (visual analogue scale) - State anxiety (STAI short) - Itch and treatment satisfaction (ecological momentary assessment) |
| t10 (Visit 9) | - State anxiety and depression (STADI-state) - Depression (BDI-II) - GEEE Abschluss - Skin-related quality of life (DLQI) - Itch (visual analogue scale) - Side effects (GASE) - PDI - Warmth and competence - Acute treatment effects (GEEE, PGA) - Expected skin-related quality of life after treatment (DLQI) - Expected symptom severity after treatment (PGA) - Subjective predictors for acute treatment effects - Stress (visual analogue scale) - State anxiety (STAI short) - PBI - Patient´s Perception on the conditioned stimulus - Itch and treatment satisfaction (ecological momentary assessment) |

Abbreviations: BDI: Beck Depression Inventory 2 (Kühner et al., 2006); BFI-10: Big Five-Inventory 10 (Rammstedt & John, 2007); DLQI: Dermatology Life Quality Index (Finlay & Khan, 1994); GASE: General Assessment of Side Effects (Rief et al. 2011); GEEE: Generic Rating Scale for Previous Treatment Experiences, Treatment Expectations, and Treatment Effects (Rief et al., 2021); ISAQ: Interoceptive Sensibility and Awareness Questionnaire (Bogaerts et al., 2022);PBI: Patient Benefit Index (Feuerhahn et al., 2012), PDI: Pain Disability Index (Dillmann et al., 2011); PGA: Patient Global Assessment (Langley et al., 2013); PSS: Perceived Stress Scale (Klein et al., 2016); SSAS: Somatosensory Amplification Scale (Barsky et al., 1990); STADI-state: state subscale of the State- Trait Anxiety and Depression Inventory (Laux et al.,2013); STADI-Trait: trait subscale of the State-Trait Anxiety and Depression Inventory (Laux et al., 2013); STAI-state: state subscale of the State-Trait Anxiety Inventory (Spielberger, 1970); TEX-Q: Treatment Expectation Questionnaire (Shedden-Mora et al., 2023); W&C: warmth and competence of the physician (Seewald et al., 2022).
