# Supplementary material for: Study protocol for testing pharmacological conditioning as a drug dose reduction strategy in patients with psoriasis in a randomised controlled trial
Source: BMJ Open. 2026 Apr 15;16(4):e114026. doi: 10.1136/bmjopen-2025-114026 (PMC13084905; doi:10.1136/bmjopen-2025-114026)
Supplement: online supplemental file 2 [file bmjopen-16-4-s002.docx]

**Supplementary Text 1:** Original German Script of the Medical Educational Video

“Liebe Patientinnen und Patienten,

Mein Name ist Dr. Frederik Krefting. Bei Ihrem letzten Besuch, haben Sie in der Patientenaufklärung ja bereits einige Informationen zur Studie bekommen. Vielleicht wissen Sie noch, dass manche Personen zusätzlich zum Medikament ein besonderes Getränk erhalten. Sie gehören zu diesen Personen und ich möchte Ihnen gerne einmal genauer erklären, was es mit dem Getränk auf sich hat.
Wir wissen schon seit längerem, dass bei der Schuppenflechte das Immunsystem - also unser körpereigenes Abwehrsystem - eine ganz entscheidende Rolle spielt. Und wir wissen auch in immer mehr Details, dass das Immunsystem eng mit unserem Gehirn verbunden ist und sich beide Seiten stetig austauschen. Sie kennen das ja vielleicht auch von Ihrer Schuppenflechte, wenn Sie mal viel Stress gehabt haben oder stark belastet waren, haben Sie das vielleicht auch an Veränderungen an Ihrer Haut gemerkt. An dieser Schnittstelle zwischen Gehirn und Immunsystem wird derzeit viel geforscht, mit dem Ziel, diese Verbindung zu nutzen und so die Behandlungen stetig zu verbessern. So wurde in der Vergangenheit in Studien gezeigt, dass man das Immunsystem nicht nur durch Medikamente, sondern auch durch Konditionierung oder Lernprozesse positiv beeinflussen kann.
Konditionierung - was war das eigentlich noch gleich? Vielleicht kennen Sie ja bereits das Experiment mit dem Pavlov´schen Hund. Immer, wenn die Hunde Futter bekommen haben, hat Pavlov eine Glocke geklingelt. Nach einer Weile hatten die Hunde gelernt, dass Glocke und Futter zusammenhängen und die Glocke allein konnte den Speichelfluss der Hunde hervorrufen. Auf die gleiche Art lässt sich auch die Gehirn-Immun-Achse trainieren, wodurch man sozusagen die körpereigene Apotheke aktiviert. Nur dass wir keine Glocke und Futter benutzen, sondern unser Getränk und das Medikament, um die Wirkung der Medikamente zu steigern.
Wichtig für die Studie ist, Sie wissen, dass Sie das besondere Getränk bekommen und die Person, die es Ihnen bringt, weiß das auch, aber der Arzt, der sie behandelt ist verblindet. Er weiß also nicht, welche Behandlung Sie bekommen. Das ist wichtig, damit sichergestellt ist, dass er die Symptome objektiv einschätzen kann und die Studie nicht verfälscht. Erzählen Sie ihm also bitte nichts davon, dass Sie das Getränk bekommen. Falls Sie noch Fragen dazu haben, wenden Sie sich gerne an die anderen Personen des Studienteams.
Ich bedanke mich im Namen meiner Kolleginnen und Kollegen nochmals recht herzlich, dass Sie an unserer Studie teilnehmen und wünsche Ihnen alles Gute für den Therapiestart.“

**Supplementary Text 2:** English Translation of Script for the Medical Educational Video

“Dear patients,

My name is Dr. Frederik Krefting. During your last visit, you already received some information about the study in the patient information leaflet. You may remember that some people receive a special dring in addition to their medication. You are one of these people and I would like to explain to you in more detail what this drink is all about.

We have known for some time that the immune system – our body´s own defense system – plays a crucial role in psoriasis. And we also know in more and more details that the immune system is closely connected to our brain and that both sides are constantly exchanging information. You may be familiar with this from your psoriasis; if you have been under a lot of stress or have been under a lot of strain, you may have noticed this in changes to your skin. A lot of research is currently being carried out on this interface between brain and the immune system, with the aim of using this connection to constantly improve treatments. In the past, studies have shown that the immune system can be positively influenced not only through medication, but also through conditioning or learning processes.

Conditioning – what was that again? You may already be familiar with the experiment with Pavlov´s dog. Whenever the dogs were given food, Pavlov rang a bell. After a while, the dogs had learned that the bell and food were connected and the bell alone could trigger the dog´s salivation. The brain-immune axis can also be trained in the same way, activating the body´s own pharmacy, so to speak. The only difference is that we don´t use a bell and food, but our drink and the medication to increase the effect of the medication.

The important thing for the study is that you know that you are getting the special drink and the person who brings it to you also knows this, but the doctor who treats you is blinded. So he does not know what treatment you are getting. This is important to ensure that he can assess the symptoms objectively and not falsify the study. So please do not tell him that you are receiving the drink. If you have any further questions, please do not hesitate to contact the other members of the study team. On behalf of my colleagues, I would like to thank you once again for taking part in our study and wish you all the best for the start of your therapy.”
