# Supplementary material for: Study protocol for testing pharmacological conditioning as a drug dose reduction strategy in patients with psoriasis in a randomised controlled trial
Source: BMJ Open. 2026 Apr 15;16(4):e114026. doi: 10.1136/bmjopen-2025-114026 (PMC13084905; doi:10.1136/bmjopen-2025-114026)
Supplement: Supplementary file 4 [file bmjopen-2025-114026.reviewer_comments.pdf]

PEER REVIEW HISTORY

BMJ Open publishes all reviews undertaken for accepted manuscripts. Reviewers are asked to complete a checklist review form and are provided with free text boxes to elaborate on their assessment. These free text comments are reproduced below.

ARTICLE DETAILS

Title (Provisional)

A study protocol for testing pharmacological conditioning as a drug dose reduction strategy in patients with psoriasis in a randomized controlled trial

Authors

Savvidou, Paraskevi; Hölsken, Stefanie; Krefting, Frederik; Benson, Sven; Sondermann, Wiebke

---

VERSION 1 - REVIEW

Reviewer 1

Name Coker, Bola

Affiliation King's College London, Primary Care And Public Health Sciences

Date 07-Jan-2026

COI None

More information is needed on the power calculation, for instance, the proportion of participants expected to have a favourable outcome in both the TAU and reinforcement groups, to allow recomputation of the sample size using the R epiR package and the numbers to be recruited based on the 2:1:1 ratio and 10% attrition.

More information is needed on the process of allocation concealment. It seems it is the same research assistant that generates the randomisation schedule and does the allocation, but please clarify.

Reviewer 2

Name Sommer, Rachel

Affiliation University Medical Center Hamburg , Institute for Health Services Research in Dermatology and Nursing (IVDP)

Date 23-Jan-2026

COI None

I was very pleased to read the manuscript “A study protocol for testing pharmacological conditioning as a drug dose reduction strategy in patients with psoriasis in a randomized controlled trial”. The manuscript addresses an important topic in psoriasis care and the study is described very precisely. I do have a very few minor points only.

Outcomes

- Itch: why did you chose to use the VAS 100 instead the NRS 10, which is well established and validated for pruritus? Patients can rate their itch more easily and reliably on a 0–10 scale. A 0–100 scale suggests a level of precision that is usually not meaningful for a subjective symptom. A 0–100 scale tends to increase variability without adding clinically relevant information; small numerical differences are rarely interpretable.

- DLQI: please add reference

PBI: Which PBI version are you planning to use? The 25-item generic version?

Other than that, I do not have any comments and I am already curious to see the exciting results.

VERSION 1 - AUTHOR RESPONSE

<u>**Reviewer 1**</u>

Comment

More information is needed on the power calculation, for instance, the proportion of participants expected to have a favorable outcome in both the TAU and reinforcement groups, to allow recomputation of the sample size using the R epiR package and the numbers to be recruited based on the 2:1:1 ratio and 10% attrition.

Response

We thank the reviewer for this helpful comment. We have expanded the description of the sample size calculation to improve transparency and reproducibility. The power calculation was conducted using the epiR package in R (function: epi.ssninfb) and was based on a non-inferiority framework using the absolute risk difference scale. For the purpose of the sample size calculation, a favorable outcome was assumed for 70% of participants in the reinforcement group and 75% in the TAU group. The non-inferiority margin was defined as an absolute risk difference of 0.25, corresponding to the predefined MCID (Minimal Clinically Important Difference). The calculation assumed a one-sided significance level of $\alpha = 0.05$ and a power of 80%. As the primary non-inferiority hypothesis compares TAU with the pooled reinforcement groups, the sample size calculation was based on a 1:1 allocation ratio.

Comment

More information is needed on the process of allocation concealment. It seems it is the same research assistant that generates the randomization schedule and does the allocation, but please clarify.

Response

We thank the reviewer for pointing out the need for clarification regarding allocation concealment. Additional information has been added to the study protocol to clarify the roles involved in this process. Specifically, the randomization schedule will be generated by a postdoctoral researcher who is not involved in patient care or data collection, while the research assistant will be responsible for participation allocation based on this schedule.

Reviewer 2

Comment

I was very pleased to read the manuscript "A study protocol for testing pharmacological conditioning as a drug dose reduction strategy in patients with psoriasis in a randomized controlled trial". The manuscript addresses an important topic in psoriasis care and the study is described very precisely. I do have a very few minor points only.

Response

We thank the reviewer for the positive and encouraging feedback on our manuscript. We are pleased that the relevance of the study was recognized. We also appreciate the reviewer´s interest in the study results.

Comment

Itch: why did you chose to use the VAS 100 instead the NRS 10, which is well established and validated for pruritus? Patients can rate their itch more easily and reliably on a 0-10 scale. A 0-100 scale suggests a level of precision that is usually not meaningful for a subjective symptom. A 0-100 scale tends to increase variability without adding clinically relevant information; small numerical differences are rarely interpretable.

Response

We thank the reviewer for this thoughtful comment. We acknowledge that the NRS (0-10) is a well-established instrument for the assessment of itch. In the present study, the VAS (0-100) was chosen to ensure consistency with the outcome assessment used across the Collaborative Research Center (CRC) 289 "Treatment Expectation", which this study is part of. This consistency allows for comparability across studies within the CRC. We will report effects sizes to avoid over-interpretation of small numerical differences.

Comment

DLQI: please add reference

Response

We thank the reviewer for pointing this out. We have added the appropriate reference for the Dermatology Life Quality Index (DLQI) in the revised manuscript.

Comment

PBI: Which PBI version are you planning to use? The 25-item generic version?

Response

We thank the reviewer for this clarification request. We will use the 25-item generic version of the Patient Benefit Index (PBI). This has now been specified in the outcome section of the revised manuscript.

Comment

Other than that, I do not have any comments and I am already curious to see the exciting results.

Response

We thank the reviewer for this encouraging remark and share the reviewer´s interest in the forthcoming results.
